# New York County

**Published:** 1896-04

**Authors:** Robt. W. Ellis

**Affiliations:** Secretary


### VETERINARY MEDICAL ASSOCIATION OF NEW YORK COUNTY.

THE regular meeting of the Association was held on March 3, 1896, at 9 o'clock P.M., with the President, Dr. Huidekoper, in the chair. On roll-call the following responded: Drs. Amling, Bretherton, C. C. Cattanach, J. S. Cattanach, Caulfield, Delaney, Ellis, Ferster, Farley, Foy, Giffin, Gill, Huidekoper, Hanson, Hueppe, Jackson, Loomes, Lamkin, Lellman, MacKellar, Neher, O'Shea, Parsons, Ryder and Turner (25).

Board of Censors (Dr. Gill, Chairman) reported that, on account of Dr. Johnson's inability to be present at the meeting of the Board, they had concluded to postpone action on his case until the next meeting, and that in the case of Turner and Finlay the Board decided to give the parties in the case until the next meeting to produce the court records, when, if they were not produced, the case would be dropped. In the case of H. Clay Glover, for violation of the Code of Ethics, the Board decided to postpone his case (he being out of the city) until next meeting.

Dr. Lellman then read a paper on "Demodex Folliculorum and Herpes in the Dog and Leukæmia in the Cat."

Dr. O'Shea, Chairman of the Committee on Legislation, reported that the Jury Bill was doing nicely, and that the Committee had received word from the Committee on Legislation of the N. Y. S. V. M. S. to the effect that an amendment had been made to the bill, introduced by Mr. C. C. Cole, of Syracuse, allowing any graduate of a veterinary school who received his degree prior to July, 1895, to register; provided he had practised veterinary medicine in some county in the State, even though he had failed to register in his own county; and that the Committee on Legislation of the County Association suggested that the applicant in the bill referred to be asked to pay $10 on presenting his application to register.

The Committee also suggested that they be empowered to have drawn up a bill requiring that all meat- and milk-inspectors in the State be graduates from veterinary schools. Moved and seconded that the report be accepted and the Committee empowered to act; carried.

Moved and seconded that the President appoint a committee of three to frame resolutions to the Fire, Police, Street-cleaning, and Charities and Corrections Departments, to the effect that they employ only graduates of veterinary colleges for the treatment of their stock; carried. This was also embodied in the work of the Committee on Legislation.

Committee on securing room at the Academy of Medicine (Dr. Hanson, Chaiman), reported that a comfortable room, seating about forty peeple, could be had for $10 a month. After some discussion it was moved and seconded that the Association take a room. An amendment to the motion was offered, to the effect that a committee be appointed to investigate the financial condition of the Association before taking a decided step in reference to securing the room. The amendment was put before the meeting; lost. The motion was then put before the meeting; carried. It was then moved and seconded that the Committee be empowered to hire the room for the next meeting; carried. Moved by Dr. Hanson, and seconded by Dr. Neher, that a vote of thanks be extended to Dr. Gill and the members of the Faculty of the N. Y. C. V. S. for their hospitality in allowing the Association the use of their rooms in the past; carried.

Moved and seconded that the meeting adjourn; carried.

ROBT. W. ELLIS, D.V.S.,
Secretary.

